# Photovoltages in Polycrystalline Mosaic Solar Cells

**DOI:** 10.1021/acsaem.1c00761

**Published:** 2021-07-14

**Authors:** S. A. Dinca, E. A. Schiff

**Affiliations:** ^†^Department of Chemistry and ^‡^Department of Physics, Syracuse University, Syracuse, New York 13244-1130, United States

**Keywords:** diffusion-limited recombination, polycrystalline thin-film solar cells, CdTe, grain boundary, minority carrier mobility

## Abstract

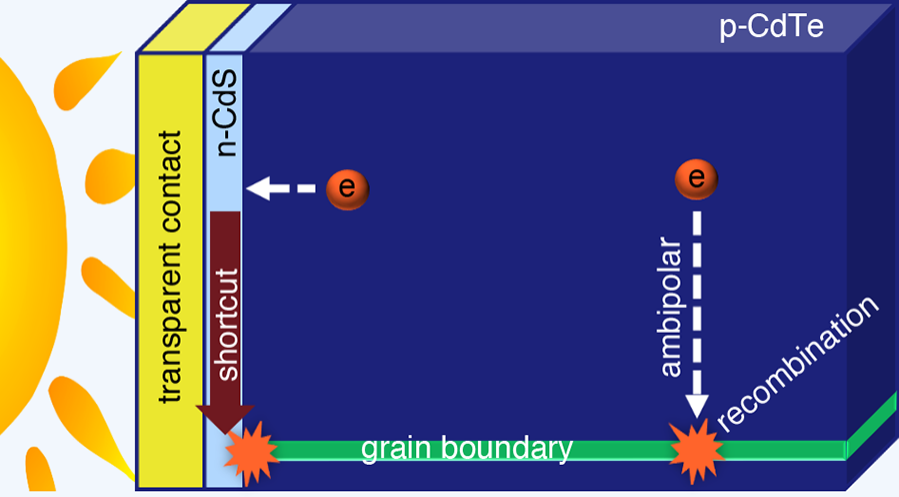

In some thin-film solar cells the light-absorbing layer is a mosaic of crystalline grains whose boundaries run from the back to the front of the cell. We used the semiconductor modeling software Sesame to do numerical calculations of the optoelectronic properties of such cells assuming that recombination of minority photocarriers occurs primarily at the grain boundaries. The work complements analytical results for diffusion-limited recombination at grain boundaries and dislocations. We chose idealized n-CdS/p-CdTe solar cells for illustration. We find that the open-circuit voltage, *V*_OC_, under illumination declines logarithmically with increasing ratio *D*/θ^2^, where *D* is the ambipolar diffusion constant governing minority-carrier transport and θ is the grain size (from 1 to 10 μm). While a decline in *V*_OC_ as mobility increases is counterintuitive, this finding is consistent with related analytical results and confirms their utility. However, open-circuit voltages are about 0.04–0.10 V lower than the corresponding analytical estimates. We show that the deficit is mostly a consequence of a recombination shortcut. At open circuit, minority photocarrier currents at points closer to the n-CdS interface than to a grain boundary are directed through the conducting front layers and terminate near the “hot spot” at the intersection with the grain boundary. The shortcut lowers open-circuit voltages by about 0.05 V below the analytical estimates.

## I Introduction

In polycrystalline semiconductors, minority photocarriers may recombine primarily at the grain boundaries. Within a grain, photocarriers generated at different locations have individual lifetimes determined by their initial distance from the grain boundary and by the ambipolar diffusion coefficient *D*. Computationally, the simplest case is a laminar stack of platelike grains with boundaries perpendicular to the cell’s surface. For plates of width θ with large grain boundary recombination velocities *S* ≫ *D*/θ, an analytical calculation for uniform photogeneration yields an average minority carrier recombination lifetime τ = θ^2^/12*D*.^1^ For cylindrical grains of diameter θ the lifetime is θ^2^/32*D* (see the Appendix). The results assume ambipolar diffusion of minority carriers and apply when the grains are not strongly depleted by charge exchange with the grain boundaries. We will discuss the other case of strong depletion in section IV. This type of “diffusion-limited” recombination model has also been formulated for dislocations threading up through a crystal.^2^ Photovoltaic cells to which analytical diffusion-limited recombination models have been applied include thin-film silicon,^3−6^ gallium nitride,^7^ halide-based perovskites,^8^ and complex organic and nanoporous materials.^9,10^ It is implicit in these models that the average photocarrier recombination lifetime τ is inversely proportional to the ambipolar diffusion coefficient and, thus, usually to the minority carrier mobility μ_min_.^11^ For the grain boundary limited lifetime just noted, no additional microscopic details of the grain boundary need to appear in the calculation.

Analytical calculations of average lifetimes are obviously useful when extended defects such as dislocations are spaced finely compared to the other relevant length scales such as the median absorption length for solar photons. For crystalline silicon, the absorption length is hundreds of micrometers.^12^ For an areal density of dislocations greater than 10^10^ m^–2^, the mean distance from a point of photogeneration to a dislocation is less than 10 μm. In such “fine-grained” materials, modeling can plausibly be done by using a one-dimensional (1D) model and the minority carrier lifetime, τ, averaged over the photogeneration profile. Thus, for uniform photogeneration and ideal contact layers, the open-circuit voltage (for a p-type material) is^4^
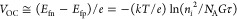
1where *E*_fn_ is defined as the mean electron quasi-Fermi level in a grain calculated from the average electron lifetime and the photogeneration rate *G*. *E*_fp_ is the hole quasi-Fermi level calculated from the acceptor concentration, *N*_A_, and neglecting the density of photogenerated holes, *p*. *n*_*i*_ is the intrinsic dark carrier density of the semiconductor at temperature *T*, *e* is the electron charge magnitude, and *kT* is the thermal energy. For average lifetimes determined by grain boundaries, we recast eq 1 using the simplest form for grain boundary recombination with laminar grains of width θ:
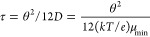
2a
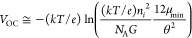
2bHere we assumed that the ambipolar diffusion coefficient is determined by the minority carrier mobility, μ_min_, and the Einstein relation: *D* = (*kT*/*e*)μ_min_. Note that increasing the minority carrier mobility decreases the open-circuit voltage; in effect, a low ambipolar diffusion coefficient “insulates” the semiconductor bulk from the grain boundaries.

In the present work, the question we ask is how well the insights from 1D models using averaged, diffusion-limited lifetimes apply to two-dimensional (2D) models of polycrystalline mosaic solar cells; we use the term “mosaic” to describe polycrystalline structures where the grain boundaries primarily run perpendicular to the surface of the cell. We chose thin-film CdTe solar cells for illustration. The champion efficiency thin-film CdTe cells have an open-circuit voltage, *V*_OC_, under solar illumination of 0.876 V at 298 K.^13^ This value is substantially smaller when compared with recent values in single crystal CdTe solar cells, where *V*_OC_ has now reached 1.096 V.^14,15^ The reduced value is plausibly attributable to grain boundary recombination.

For our numerical calculations, we used Sesame, a semiconductor modeling software developed to solve the drift-diffusion-Poisson equations in multidimensions.^16^ The material parameters are summarized in Table 1. The parameters are similar to previous modeling of large grain CdS/CdTe solar cells^17−23^ but were selected to avoid substantial charge depletion of the grains by the grain boundaries.

**Table 1:** List of Parameters Used in the Numerical Simulations

parameters	CdS	CdTe
thickness *x* (nm)	10	3000
grain size θ (nm)		10^3^–10^4^
energy gap *E*_g_ (eV)	2.4	1.5
electron affinity χ (eV)	4.38	4.28
dielectric constant ε	8.4	10.2
acceptor concentration *N*_A_ (cm^–3^)	0	1.0 × 10^15^
donor concentration *N*_D_ (cm^–3^)	1.0 × 10^17^	0
electron mobility μ_e_ (cm^2^/(V s))	160	9–1000
electron lifetime τ_e_ (ns)	0.1	see *L*_D_
hole mobility μ_h_ (cm^2^/(V s))	15	80
hole lifetime τ_e_ (ns)	10^–3^	see *L*_D_
ambipolar diffusion length *L*_D_ (cm)^34^		>θ

CdTe grain boundary parameters	
defect type	donor (D)
defect density per area *N*_gb_ (cm^–2^)	1.0 × 10^10^
defect energy level *E*_t_ (eV)	0.75 eV below *E*_C_^a^
capture cross section for electrons σ_e_ (cm^2^)	1.0 × 10^–11^
capture cross section for holes σ_h_ (cm^2^)	1.0 × 10^–12^

a *E*_C_ denotes the energy of the conduction band minimum.

**Figure 1:**
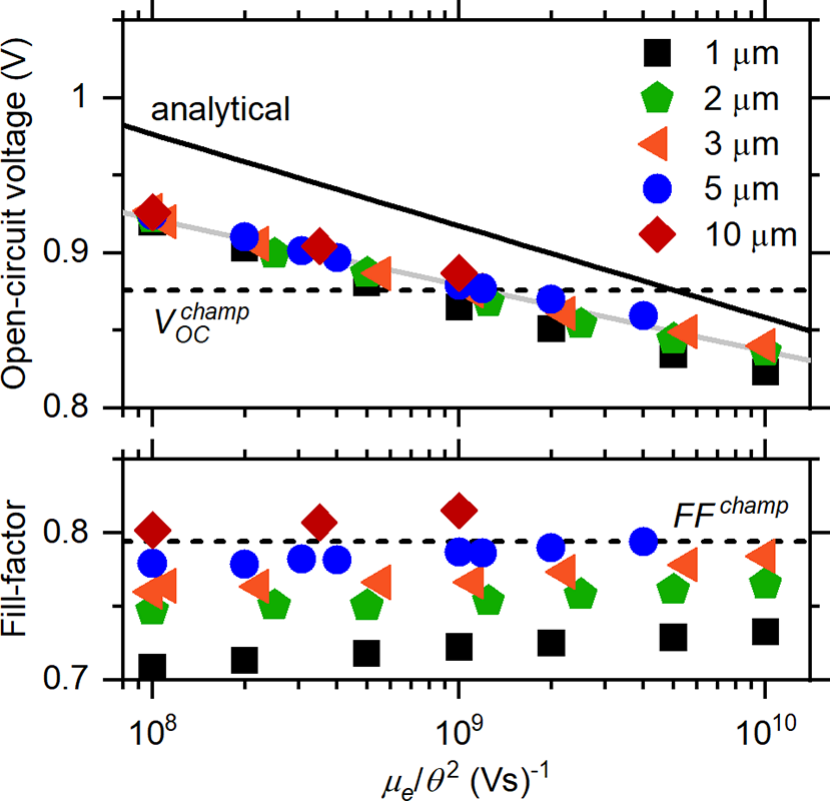
Symbols indicate numerical calculations for the open-circuit voltages *V*_OC_ (upper panel) and the fill factors *FF* (lower panel) for CdS/CdTe solar cells under solar illumination at 300 K. The horizontal axis is the ratio of electron carrier mobility μ_e_ and the square of grain size θ as given in the key. Note the linear dependence of the *V*_OC_ upon the μ_e_/θ^2^ ratio as guided by the solid gray line. The solid line represents the analytical solution given by eq 2b with uniform photogeneration *G* = 6.3 × 10^20^ cm^–3^ s^–1^. The dashed lines are guides for the eye and indicate the open-circuit voltage *V*_OC_^champ^ and the fill factor *FF^champ^* for the champion CdTe thin-film solar cell at 298 K.^13^

The symbols in the upper panel of Figure 1 show the dependence of the numerical calculations of *V*_OC_ upon the ratio μ_e_/θ^2^, where electrons are assumed to be the minority carrier and μ_e_ is their mobility. The calculations are done under standard AM 1.5G 1-sun illumination conditions (100 mW/cm^2^) at 300 K. The solid line labeled “analytical” is derived from eq 2b and assumes uniform photogeneration (*G* = 6.3 × 10^20^ cm^–3^ s^–1^) yielding the same short-circuit current density, *J*_SC_, as for solar illumination conditions. The numerical and analytical results have essentially the same dependence on μ_e_/θ^2^. As we discuss subsequently, the decrease of about 0.05 V between the analytical and numerical results is not primarily due to the difference in solar and uniform photogeneration profiles. It is mostly due to a minority photocarrier shortcut to recombination through the CdS emitter and the front contact layers; the actual recombination event is near the intersection of a grain boundary and the CdS layer. We call this recombination region near the intersection a “hot spot”. Such hot spots were noted by Gaury and Haney in CdTe cell simulations^24^ and in much earlier work in polycrystalline silicon.^25^

The numerical calculations of the fill factors *FF* for the cells have a quite different dependence on the electron carrier mobility, μ_e_, and grain size, θ. To our knowledge, there have not been related analytical calculations. There is only a slight increase in *FF* with μ_e_, which contrasts with the decrease in *V*_OC_. We think this is because of the very small depth of solar photogeneration in CdTe (∼0.3 μm), which avoids photoinduced space-charge effects.^26^ The noticeable increase in *FF* with grain size is a second consequence of the shortcut to the recombination hot spot. The effect is qualitatively similar to interface recombination at the CdS/CdTe interface but intrinsically varies with grain boundary properties.

For reference, we also show the values of the open-circuit voltage, *V*_OC_^champ^, and the fill factor, **FF**^*champ*^, for the present champion thin-film CdTe solar cell (see dashed lines in Figure 1).^13^ The champion open-circuit voltage, *V*_OC_^champ^, corresponds to μ_e_/θ^2^ = 10^9^ V^–1^ s^–1^. To achieve the single-crystal cell *V*_OC_ ≈ 1.10 V in a polycrystalline cell would require μ_e_/θ^2^ = 10^4^ V^–1^ s^–1^, which would correspond to grain sizes of hundreds of micrometers. At the same time, the ambipolar diffusion length, *L*_D_, within a grain would need to be larger than the grain size, θ—otherwise, the bulk recombination of the intragrain material would limit the open-circuit voltage, which would become independent of the grain size. A simulation for 100 μm grains, an electron carrier mobility of 100 cm^2^/(V s), and a correspondingly enormous recombination time (τ = 2 × 10^–4^ s) gave a *V*_OC_ = 1.02 V, which is on the (solid gray) trend line of Figure 1.

The trade-off of the electron carrier mobility and the grain size does not apply to the cell fill factor, which increases mostly with grain size, θ. Note, a grain size θ = 5 μm gave a fill factor as large as the champion cell. We note that the analytical calculations presented in the Appendix show that laminar grains lead to *V*_OC_ values about 0.02 V higher than for cylindrical grains. Cylindrical grains would arguably be a better approximation to actual polycrystalline mosaic cells than laminar grains.

In the following, we first present the details of the numerical solar cell calculations summarized in Figure 1. We then show the complex contour plots of photocarrier densities and currents from the simulations. Inspecting these plots, it is not obvious why the analytical average lifetime approach is a useful guide to *V*_OC_. It turns out that much of this complexity is associated with the intersection of a grain boundary and the front, n-type CdS layer. As noted earlier, near the intersection, there is a recombination hot spot that is not envisioned in the analytical calculations. Here, we show that electrons generated near the CdS/CdTe interface, but relatively distant from the grain boundary, induce an Ohmic current of electrons in the CdS and the front contact layers that terminates at the hot spot. We identify this recombination shortcut as a channel that lowers the open-circuit voltage. A minority photocarrier at a particular point in the CdTe that is closer to the CdS than to the grain boundary needs to diffuse only the shorter distance before merging into a high conductivity highway to the hot spot. As we illustrate in section III, this recombination shortcut lowers *V*_OC_ by about 0.05 V. This recombination channel would mimic true interface recombination in experiments and possibly account for the improvement in fill factors with grain size in Figure 1.

## II Simulation Details

**Figure 2:**
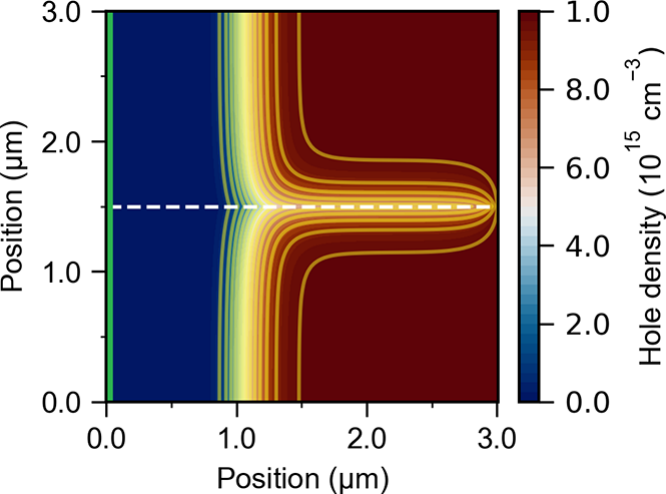
Contour plot for the hole density of the CdS/CdTe cell with a single planar grain boundary (dashed line) perpendicular to the junction, under short-circuit conditions in the dark. The thin green layer indicates the 10 nm thick n-type CdS emitter layer. The built-in potential is *V*_BI_ = 1.08 V, the acceptor concentration is *N*_A_ = 1.00 × 10^15^ cm^–3^, and the grain-boundary donor density is *N*_gb_ = 1.00 × 10^10^ cm^–2^. The thickness of the depletion layer near the CdS layer and that of the depletion region of the grain boundary (solid yellow contour lines) agree with elementary calculations.

The numerical simulations were performed by using the two-dimensional (2D) semiconductor modeling software Sesame to solve the electron and hole continuity equations simultaneous with the Poisson equation.^16^ The script calculates the properties of a pn^+^ junction solar cell with a polycrystalline array of grains with planar grain boundaries. For orientation, in Figure 2 we show the contour map of the hole density calculated under dark conditions at 0 V for the CdS/CdTe solar cell model system. A single planar grain boundary is positioned in the middle of the CdTe absorber, perpendicular to the junction between CdS and CdTe layers, and terminates at 0.05 μm from the p-type (right) contact. The grain boundary extends indefinitely down into and up out of the page. The grain width is θ = 3 μm, which is also the thickness of the p-type CdTe absorber layer. The model assumes that the incident light enters through the front n-type CdS emitter (window) layer, the thin green layer at the left in Figure 2. The solar photogeneration profile is calculated by using Sesame’s built-in table for the 1-sun terrestrial illumination (100 mW/cm^2^ AM1.5G) spectrum^27^ and those for the absorption spectra of CdS and CdTe layers.^28,29^ The numerical integration of the resulting generation rate profile, *G*(*x*), yielded a short-circuit density current of about 28.5 mA/cm^2^. As other workers have noted, the champion cell’s short-circuit current is about 2 mA/cm^2^ larger than can be explained by optical absorption measurements on CdTe;^20,30^ the short-circuit current density *J*_SC_ reported for the champion cell was 30.25 mA/cm^2^.^13^ We assumed selective Ohmic contacts such that electron current vanishes at *x* = *L* (right contact) and the hole current vanishes at *x* = 0 (left contact).

The assumption of a laminar array of grains is computationally and analytically convenient; however, it is a rough approximation for a mosaic of columnar grains. The corresponding analytical model noted earlier yields an average steady-state recombination lifetime for photocarriers in a grain of τ = θ^2^/12*D* with large grain boundary recombination velocities. In the Appendix, we show that a cylindrical grain geometry yield τ = θ^2^/32*D*. The laminar approximation for *V*_OC_ is then larger than for the cylindrical geometry by (*k*_B_*T*/*e*) ln(8/3) = 0.026 V.

The acceptor concentration of the CdTe layer was set to *N*_A_ = 10^15^ cm^–3^. This value is in the range of previous modeling of CdTe solar cells and is large enough that the photogenerated density of minority carriers remained smaller than *N*_A_.^20,24,31^ We assumed the planar grain boundary has a donor density *N*_gb_ = 10^10^ cm^–2^. This small donor density was chosen to clarify comparison with the diffusion-limited model even for 1 μm grains. Generally, we expect the model to apply when the grain boundary depletion width is much smaller than the grain width θ. Our assumptions for the grain boundary parameters correspond to a very thin depletion layer (0.1 μm) extending into the CdTe layer. The depletion regions to the right of the CdS interface and above and below the grain boundary are evident in Figure 2.

In Table 1, we present the primary parameters used in the simulations; some additional parameters normally used for CdTe and CdS are given in an endnote.^32^ We varied the minority carrier (electron) mobility μ_e_ widely, from 9 to 1000 cm^2^/(V s). The bulk ambipolar diffusion length of the photocarriers, *L*_D_, in the p-type CdTe layer was kept larger than the width of the grains, such that the calculations were focused on recombination at the grain boundaries. The grain boundary recombination velocity for electrons *S* = *N*_gb_σ_e_*v*_th_ was set to be 10^6^ cm/s, which is near the upper range of experimental estimates in p-type CdTe and previous modeling studies,^17,33^ where *v*_th_ = 10^7^ cm/s is the thermal velocity and σ_e_ is the capture cross section for electrons. This corresponds to a fairly large electron capture cross section, σ_e_ = 1.0 × 10^11^ cm^2^. The full analytical formula for the effective recombination lifetime of a polycrystalline bulk material with diffusion-limited recombination at planar surfaces is^1^
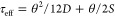
3The diffusion and recombination velocity terms are comparable only at the extremes of our simulations (θ = 10^–4^ cm, *D* = 25 cm^2^/s). If the recombination velocity is small and its term in eq 3 is larger than the diffusion term, the minority photocarrier density will vary little within a grain, and the ambipolar diffusion coefficient (and hence the minority carrier mobility) will not affect the average lifetime or the open-circuit voltage. Given the presumption of a large grain boundary recombination velocity and neglecting true interface recombination at the CdS/CdTe interface, only three materials quality parameters significantly affected the simulations: the CdTe acceptor concentration *N*_A_, the grain size θ, and the electron mobility μ_e_. The numerical simulation was relatively insensitive to most of the other parameters: the density of grain boundary donors *N*_D_, the electron capture cross sections σ_e_, the hole capture cross section σ_h_, the majority (hole) carrier mobility μ_h_, and the bulk ambipolar diffusion length *L*_D_ (as long as it is larger than θ).

## III Dependence of *V*_OC_ on the Ratio μ_e_/θ^2^

**Figure 3:**
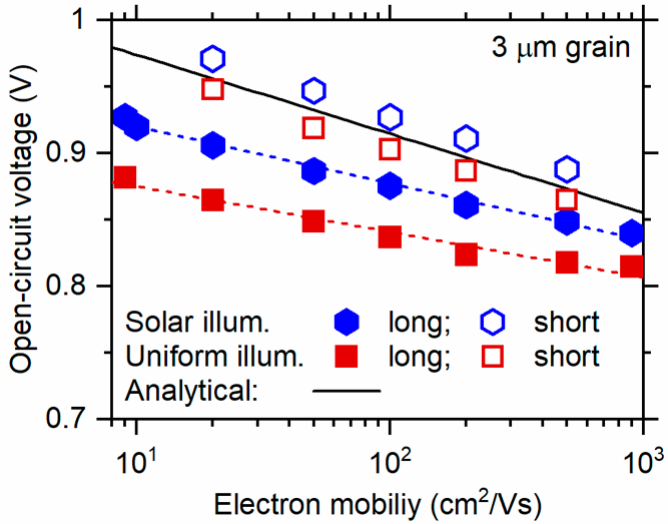
Analytical and numerical estimates of the open-circuit voltage, *V*_OC_, for cells with θ = 3 μm thick grains and varying electron carrier mobilities, μ_e_. The blue (open and solid) symbols indicate *V*_OC_ under solar illumination. The red (open and solid) symbols and the “analytical” line are obtained with uniform photogeneration (*G* = 6.3 × 10^20^ cm^–3^ s^–1^). The open symbols are results obtained with a shortened grain boundary that stops 0.4 μm before the CdS/CdTe interface. The dashed red and blue lines are guides only.

We return to the dependence of the open-circuit voltage upon the ratio μ_e_/θ^2^. A material with a perfect junction and perfect contacts, under uniform photogeneration, *G*, and sufficiently large grain-boundary recombination velocity should obey eq 2 for its implied open-circuit voltage, *V*_OC_. In Figure 3, we graph this analytical calculation (solid black line) along with the numerical calculations for both solar illumination and uniform photogeneration with the same absorbed photon flux (*G* = 6.3 × 10^20^ cm^–3^ s^–1^) in the CdTe layer. The solid symbols are calculated with a grain boundary that runs from 0.05 μm above the p-type back contact to the CdS/CdTe interface. The open symbols are a simulation in which the grain boundary ends 0.4 μm below the CdS/CdTe interface.

It is unsurprising that the numerical calculations with solar illumination give larger values for *V*_OC_ than with uniform photogeneration. The solar photogeneration profile in CdTe absorber layer is strongly peaked near the CdS interface, and the electron quasi-Fermi level near that interface determines *V*_OC_.^26,35^ With the full-length grain boundary, there is a deficit of around 0.05 V between the analytical and numerical calculations with uniform photogeneration. As we shall illustrate shortly, this deficit is mostly due to the recombination shortcut for electrons which terminates at the intersection of the grain boundary and the CdS/CdTe interface. When the grain boundary terminates short of the interface, there is good agreement between the analytical and numerical calculations.

**Figure 4:**
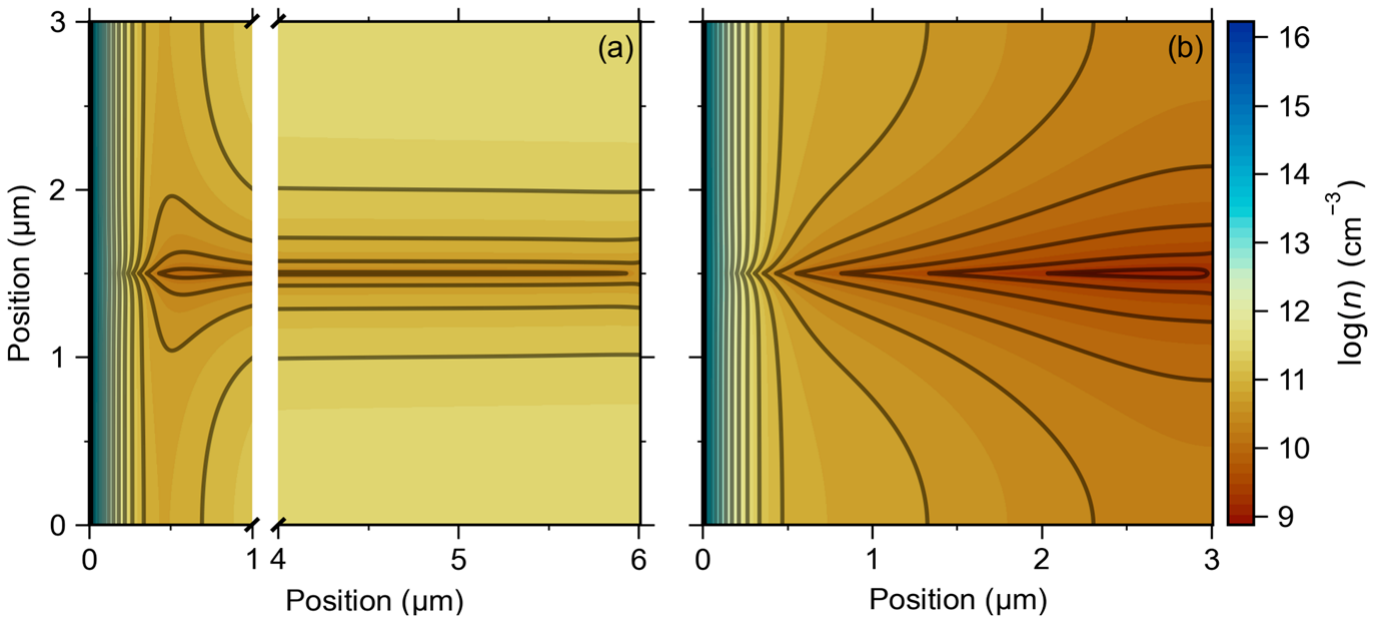
Numerical calculations of the electron density contour maps at open-circuit voltage for (a) a 6 μm thick cell under uniform photogeneration (*G* = 6.3 × 10^20^ cm^–3^ s^–1^) and (b) a 3 μm thick cell under solar illumination containing a single grain boundary intersecting the n-type CdS layer (at the left edge) at its center. The solid (dark gray) lines are guides for the reader’s eye and illustrate the electron density contours. The grain boundary contains a single donor with a defect density *N*_gb_ = 10^10^ cm^–2^ and a recombination velocity *S* = 10^6^ cm/s and is located at 0.85 eV (for uniform photogeneration) and at 0.75 eV (for solar illumination) below the conduction band edge, respectively. All calculations are done for μ_e_ = 100 cm^2^/(V s) and μ_h_ = 80 cm^2^/(V s); the simulation parameters are given in Table 1.

We explore the numerical calculations further in Figure 4, which uses μ_e_ = 100 cm^2^/(V s) and a full-length grain boundary. Figure 4a shows the electron density contours for a very thick cell (*x* = 6 μm) and uniform photogeneration. Deep in the CdTe layer, the contours correspond well to the diffusion-limited, analytical calculation. The maximum electron density is in a plane separated by 1.5 μm from the grain boundary planes; the density declines parabolically to near zero at the grain boundary. Near the CdS/CdTe interface, the contours are complex as they go from perpendicular to parallel to the interface. Given the complexity, it is remarkable that the analytical scaling of *V*_OC_ with −(*kT*/*e*) ln(μ_e_/θ^2^) still applies fairly well. Figure 4b shows the electron density contours with solar illumination for a 3 μm cell. The complexity of the electron density contours increases further because the photogeneration is close to the CdS interface instead of being spread uniformly through the cell. As presented in Figure 3, solar illumination increases *V*_OC_ from 0.84 to 0.88 V, and its scaling with −(*kT*/*e*) ln(μ_e_/θ^2^) has the same trend as with uniform photogeneration.

**Figure 5:**
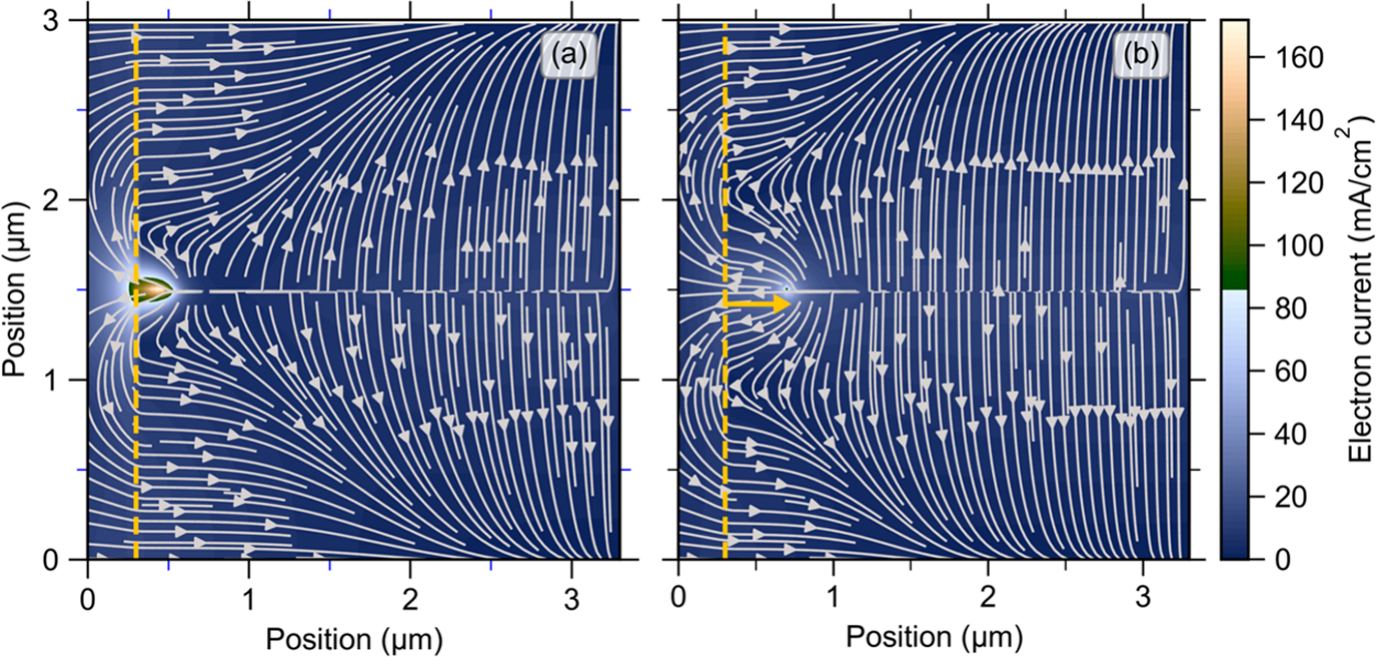
Electrical current flows of electrons at open circuit with uniform photogeneration (*G* = 6.3 × 10^20^ cm^–3^ s^–1^) for two types of grain boundary lengths: (a) a full-length grain terminated at the CdS interface and (b) a shorter grain terminated 0.4 μm before it reached the CdS layer interface; the orange arrow indicates the position at which the grain boundary terminates. The dashed orange lines mark the CdS/CdTe interface. The CdS layer thickness was increased to 0.3 μm for a better illustration of the electron current flow. The gray arrows indicate the direction of the electron flow. The numerical calculations are done for μ_e_ = 100 cm^2^/(V s) and μ_h_ = 80 cm^2^/(V s).

One physical reason for why the analytical result of eq 1 does not give a precise account for the magnitude of *V*_OC_ in cells with uniform photogeneration proves to be simple. In Figure 5a, we show a map of calculated electron currents at open-circuit conditions and with uniform photogeneration. For illustration only, we made the CdS layer 0.3 μm thick. Electrons near the back of the cell flow in the expected pattern toward the planar grain boundary and recombination. On the other hand, electrons closer to the front CdS interface flow first into the highly conducting CdS. As illustrated in Figure 5a, the electron current continues through the CdS and front contact. It finally delivers electrons to the hot spot where they recombine with holes. Once a photogenerated electron reaches the CdS layer, the distant recombination event at the hot spot occurs nearly instantaneously due to dielectric relaxation processes. We refer to this recombination channel as the “shortcut” channel. This effect is further illustrated in Figure 5b, where the grain boundary was terminated about 0.4 μm from the CdS interface. The shortening of the grain reduces the effect of the shortcut channel. Electron currents from the CdS to the hot spot now need to surmount the barrier presented by the depletion region to reach the hot spot. The hot spot is noticeably cooler, and *V*_oc_ increased, as shown in Figure 3.

## IV Discussion

The model and simulations in this study were restricted to the regime of minimal depletion of the intragrain material by grain boundary defects. Specifically, we chose a grain boundary donor density, which corresponds to a depletion layer (0.1 μm) that was much less than the grain width, θ, in all studied cases, as illustrated in Figure 2. Recent measurements of the depletion potential between grain boundaries and intragrain material in polycrystalline CdTe show substantial variability, but the typical order is 0.1 V.^36^ This potential corresponds to a 0.3 μm depletion width in the CdTe absorber, which would have only a small effect on the present calculations.

**Figure 6:**
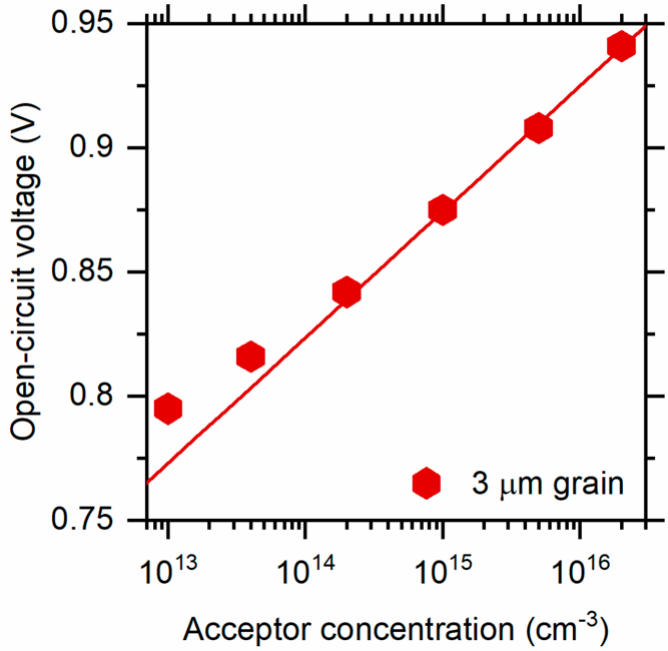
Dependence of the open-circuit voltage *V*_OC_ (solar illumination) on the CdTe acceptor concentration *N*_A_. Symbols are numerical results obtained with an electron carrier mobility μ_e_ = 100 cm^2^/(V s) and 3 μm grain widths; the solid line is a least-squares fit to the three highest values and yields a slope of +50 mV/decade. The material model predicts a slope of +58 mV/decade at 300 K.

The contrasting case of substantial depletion of the intragrain material of larger densities of grain boundary defects (1.0 × 10^14^ cm^–2^ instead of 1.0 × 10^10^ cm^–2^ in the present case) has been well-studied by Gaury and Haney.^17^ Despite the enormous difference in the grain boundary densities, their model predicts a similar functional dependence of the open-circuit voltage, *V*_OC_, upon the minority carrier mobility, μ_e_, to those presented in Figure 4. An important difference between the two regimes is the dependence of the photovoltage on the CdTe acceptor density *N*_A_. We illustrate the results for the present simulations in Figure 6 for a 3 μm grain and an electron carrier mobility μ_e_ = 100 cm^2^/(V s). Equation 2b predicts the dependence Δ*V*_OC_ = (*kT*/*e*)Δ ln *N*_a_, which corresponds to a slope of +58 mV/decade at 300 K. The trend line, in Figure 6, shown has a slope of +50 mV/decade. The agreement seems adequate considering the neglect of the doping-dependent depletion region near the CdS/CdTe interface. However, in ref (17), when the ambipolar diffusion length was longer than the grain width (see Figure 8d in ref (17)), the sign of the doping effect is reversed from our results. Interestingly, when the diffusion length becomes comparable to the grain width, the dependence of *V*_OC_ on *N*_A_ has an inverted U-shape, with a maximum value near 3.0 × 10^15^ cm^–3^.

The two-dimensional simulations of Kanevce *et al.*(20) assumed negligible intragrain depletion, as we have here. Their simulations used μ_e_ = 320 cm^2^/V throughout but varied the grain boundary recombination velocity widely. Where there is overlap, their simulations agree well with the present simulations. This work also studied the effects of interface recombination at the CdS/CdTe interface, which reflects experiments consistent with this process. In experiments, the recombination shortcut to hot spots that we have identified in the present work would be difficult to distinguish from true interface recombination. The shortcut recombination mechanism has a significant dependence on the grain size as suggested by the lower panel of Figure 1. True interface recombination at the CdS/CdTe interface adds an additional recombination mechanism that would be independent of grain size. In cells with the grain boundary shortcut, materials processing that eliminates or passivates grain boundaries near the front junction could improve photovoltages by as much as 0.05 V. A difficulty is that extended defects such as dislocations and grain boundaries are difficult to terminate. It is possible that techniques for processing the CdS/CdTe interface, which are known to affect apparent interface recombination, are already passivating the near-interface region of CdTe.^37^ Recent technologies have improved the efficiency of CdS/CdTe cells by alloying CdTe with selenium (Se) near its interface with CdS. A study by Fiducia *et al.*(38) concludes that Se accumulates near the grain boundaries and passivates intragrain defects.

## V Conclusion

We compared an elementary model for the photovoltages obtainable from a polycrystalline material with numerical simulations of CdS/CdTe solar cells. The grain boundaries run through the p-type CdTe layer and perpendicular to its surface. Photocarrier recombination occurs at the grain boundaries and is diffusion-limited. The simulations incorporate device interfaces that might confound the elementary model, which predicts that the photovoltage should decline as −(*kT*/*e*) ln(μ_min_/θ^2^). Our major findings are the following:

- The elementary absorber material model predicts the photovoltage in simulated cells well for a large range of μ_min_ and θ. However, photovoltages from cell simulations are lower than the material model by a deficit of about 0.05–0.10 V.
- The deficit is due to a shortcut for minority carrier photocurrents. The shortcut leads through the CdS to reach a recombination hot spot near the intersections with the grain boundaries. Experiments that are typically interpreted in terms of interface recombination would also be sensitive to the shortcut recombination mechanism.
- For simplicity, grains that are parallel thin slabs running perpendicular to the cell surface are typically used in numerical simulations. The analytical material model was also solved here for cylindrical grains. For large grain boundary recombination velocities, cylindrical grains yield open-circuit voltages that are (*kT*/*e*) ln(8/3) smaller than with slab grains.

These results indicate the importance of passivation of the near-interface grain boundaries even in materials with low densities of intragrain recombination centers. In addition, the results presented here suggest at least two directions for future research in CdTe and other polycrystalline mosaic solar cells. The recombination hot spot at the grain boundary/emitter interface is responsible for a significant loss in cell efficiency. Research directed explicitly at visualizing, understanding, and passivating the hot spots could be productive. There may also be engineering strategies for the emitter/absorber interface region that would reduce their effect. Second, minority carrier mobility engineering is an opportunity for improved efficiency in the thin-film polycrystalline cells. The mobilities need to be as small as possible to build up the intragrain photocarrier concentration under illumination in the presence of grain boundary recombination but large enough to keep intragrain recombination under control. Direct measurements of carrier mobilities, in particular for thin-film CdTe, would be a useful counterpart for time-resolved photoluminescence and other indirect measurements.

## Appendix. Diffusion-Limited Recombination in Cylindrical Columns

In a doped polycrystalline semiconductor under illumination, photocarrier transport in most regions is due to ambipolar diffusion of the minority carriers. For a p-type semiconductor, the minority carriers are electrons, and their density *n*(*x*,*y*,*z*) at steady-state can be described:

A1

where *D* is the ambipolar diffusion coefficient for the carriers and *G* is the photogeneration rate, which we assume to be uniform. A simple case is a slab crystallite of thickness θ; Wight *et al.* provided the solution:^1^

A2

where *S* is the recombination velocity at the grain boundaries (*x* = θ/2 and *x* = −θ/2) and the corresponding boundary condition at *x* = θ/2 is . This is the geometry assumed by the Sesame code that we have used for the numerical calculations in the present paper. The average lifetime of the electron photocarriers is defined by using the average electron photocarrier density:

A3

This yields the solution noted earlier:

A4

For a cylindrical grain of diameter θ, the fundamental equation with cylindrical coordinates and symmetry is

A5

where ρ is the distance of a point from the cylinder axis. With the boundary condition  at ρ = θ/2, the solution for the density is

A6

This leads to

A7

For large recombination velocities, the assumption of slab grains geometry average yields lifetimes longer by 8/3 than assuming cylindrical grains. With small recombination velocities, the increase is 2-fold. The average lifetimes are converted to open-circuit voltages by using eq 1.
